# Mechanistic Study on Nb Microalloying Synergistically Enhancing the Cryogenic Strength–Toughness of Austenitic Stainless Steel

**DOI:** 10.3390/ma19214719

**Published:** 2026-11-06

**Authors:** Lei Huang, Riqing Huang, Qing Li, Xiangzhu Wu, Tingping Hou, Tianliang Zhao, Sundas Arshad, Marina Afzal, Faiqa Barkat, Kaiming Wu

**Affiliations:** 1 Guangxi BG New Materials Co., Ltd., Beihai 536000, China; 2 School of Metallurgy and Energy, International Research Institute for Steel Technology, Wuhan University of Science and Technology, Wuhan 430081, Chinaustb_tlzhao@163.com (T.Z.);

**Keywords:** 304 austenitic stainless steel, Nb microalloying, cryogenic temperature, tensile properties, impact properties

## Abstract

Overcoming the strength–ductility trade-off at cryogenic temperatures is a key bottleneck for austenitic stainless steels. This study investigated the effects of Nb additions (0, 0.03, and 0.06 wt.%) on the mechanical properties and fracture behavior of 304 steel from room temperature (RT) to −196 °C. Within the investigated range, the 0.06 wt.% Nb steel produced the largest measured response. Compared with the Nb-free steel, the RT yield strength and elongation after fracture increased by ~5% and 2.4%, respectively; at −196 °C, they increased by 11.1% and 4.1%, and impact toughness by 31.8%, mitigating the low-temperature strength–ductility trade-off. Fractography revealed a transition from brittle cleavage/mixed fracture to dimple-dominated ductile fracture. The improvement in mechanical properties is consistent with the refined microstructure and the modified inclusion distribution, with the underlying mechanisms likely involving grain refinement and the solid-solution strengthening of the austenite matrix by Nb. It should be noted that, owing to experimental limitations, the deformation-induced α′-martensite was not quantitatively characterized in the present study, and no rigorous quantitative correlation has yet been established between inclusion characteristics and low-temperature impact toughness. Consequently, the relative contribution of each contributing factor to the observed performance enhancement remains unresolved. Nevertheless, this work provides experimental support for the microalloying design of ultra-low-temperature austenitic stainless steels.

## 1. Introduction

Austenitic stainless steels, owing to their face-centered cubic structure, exhibit exceptional cryogenic toughness [1,2,3] and have thus become indispensable materials in extreme environments involving liquid nitrogen, liquid oxygen, and liquid hydrogen [4]. With the rapid development of energy, aerospace, and physics research, increasingly stringent demands are placed on materials serving at cryogenic temperatures. In particular, applications such as liquefied natural gas (LNG) transmission pipelines, aerospace cryogenic fuel tanks, and superconducting magnet support systems require materials that not only withstand extremely low temperatures but also possess adequate strength, toughness, and corrosion resistance [5]. Although conventional austenitic stainless steels exhibit good toughness at ambient and low temperatures, their toughness reserve at cryogenic temperatures may still be insufficient to meet certain special service conditions, especially in stress-concentrated zones, weld heat-affected zones, or under complex loading states, where the materials are prone to brittle fracture risks [6,7,8].

Among the various methods for enhancing steel performance, microalloying technology has become a research hotspot due to its notable effectiveness and cost control. Among the microalloying elements, niobium (Nb) has shown great potential in optimizing the cryogenic toughness of austenitic stainless steels [9,10]. The mechanisms of Nb microalloying are multifold. First, grain refinement. Nb forms stable Nb(C,N) precipitates with C and N, which can pin grain boundaries through the Zener effect [11] and inhibit austenite grain growth during thermomechanical processing; additionally, solute Nb enhances the resistance to recrystallization by dragging grain-boundary migration. Both pathways refine the grains, and according to the Hall–Petch relationship, grain refinement can simultaneously increase strength and improve toughness [12]. Second, inclusion modification. The addition of trace Nb may alter the elemental segregation and activity conditions during solidification and deoxidation, thereby modifying the type, size, and distribution of the inclusion population; this, in turn, could improve the deformation compatibility of inclusions and reduce their role as crack nucleation sites. It must be emphasized that the EDS analysis in this study detected no Nb within the examined inclusions, indicating that under the present experimental conditions Nb did not directly participate in inclusion precipitation by forming Nb-containing inclusions. Rather, the evolution of the inclusion population is an accompanying phenomenon following Nb addition. Third, grain-boundary stabilization. Nb addition suppresses the precipitation of chromium carbides at grain boundaries [13,14], reducing intergranular corrosion susceptibility and enhancing microstructural stability at low temperatures. It should be noted that the dominant mechanism depends strongly on the C and N contents, Nb level and heat-treatment regime: Nb(C,N) precipitation usually prevails under high-carbon or aged conditions, whereas solute-drag grain refinement and inclusion modification are more prominent in low-carbon, solution-treated steels. In the present 304 stainless steel (C ≤ 0.08 wt.%), Nb exists predominantly in solid solution after solution treatment, and no distinct Nb(C,N) precipitates are observed; consequently, grain refinement and the matrix-strengthening effect of solid-solution Nb constitute the primary mechanisms addressed in this work.

Studies have shown that the improvement in cryogenic toughness of austenitic stainless steels by Nb microalloying is closely related to its addition level, existing forms, and heat treatment conditions. In AISI 347H (a Nb-stabilized austenitic stainless steel), the Nb content is controlled within a specific range (≥8 × C%) and preferentially combines with carbon to form stable NbC, which not only eliminates the risk of intergranular corrosion caused by chromium carbide (Cr_23_C_6_) precipitation at grain boundaries [15,16,17] but also maintains high impact toughness at −40 °C through grain refinement and microstructural regulation. Relevant experiments have demonstrated that Nb microalloying can significantly enhance the impact toughness of austenitic stainless steels at cryogenic temperatures and further lower the ductile–brittle transition temperature [18,19,20]. In terms of weldability, Nb-containing steels also exhibit outstanding performance, as Nb inhibits grain coarsening in the weld heat-affected zone and improves the low-temperature toughness of joints [21,22]. These results highlight the multi-mechanism synergy of Nb microalloying, with grain refinement and inclusion modification being the two aspects focused on in this study.

Although the beneficial role of Nb microalloying is well recognized, its addition level, form of existence, and synergy with heat treatment still require precise control. Excessive Nb addition may lead to the formation of coarse precipitates, thereby degrading both strength and toughness [23]. Previous studies have mainly focused on relatively high Nb contents, cryogenic toughness [9,10,11,12,13,14,15,16,17,18,19,20], or inclusion control. This study therefore provides three specific contributions: (i) a systematic investigation of the ultra-low Nb range (0–0.06 wt.%) in 304 stainless steel, which has not been systematically clarified before; (ii) a comparison, using specimens from the same heats, of the tensile properties at room temperature and −196 °C, as well as the impact toughness at room temperature, −60 °C, −120 °C, and −196 °C, revealing the non-monotonic dependence on Nb content and the strength–ductility–toughness balance; and (iii) a linkage between Nb-induced inclusion and microstructural changes and the evolution of low-temperature properties. The results show that, within the investigated range, adding 0.06 wt.% Nb achieves the optimal combination of strength, ductility, and cryogenic toughness.

## 2. Materials and Methods

The experimental materials used in this study were 304 austenitic stainless steel produced in accordance with the SUS304L (UNS S30403) standard [24], sourced from three heats within the same continuous casting sequence. Using the microalloyed Nb content as the sole design variable, three types of specimens—Nb-free, Nb-0.03, and Nb-0.06—were prepared by adding varying amounts of Nb during the LF refining stage. The specimens were designated based on their Nb mass fraction. Aside from Nb, the remaining elements were controlled to meet the SUS304L specifications; minor variations in C, Cr, Mn, Ni, and S among the three heats were considered routine compositional fluctuations typical of different heats within the same casting sequence. In this steel, Ti and Ca are tramp elements inherently associated with the lateritic nickel-ore raw material rather than deliberately added alloying elements. During smelting and solidification, these ore-associated elements participate in inclusion formation; Ca may also originate from slag-making additions. All specimens were produced on an industrial scale at Guangxi Beigang New Materials Co., Ltd. The production process flow was as follows: hot metal + medium-frequency furnace crude molten steel → AOD refining → LF refining (Nb microalloying) → continuous casting → slab reheating → rolling → black pickled coil → solution treatment → white coil. The chemical compositions of the three specimens are presented in Table 1.

All experimental samples were cut from the three types of plates (Nb-free, Nb-0.03, Nb-0.06) using wire electrical discharge machining. The specimens for microstructural observation had dimensions of 10 mm × 10 mm × 5 mm. Impact specimens were of V-notch type with dimensions of 5 mm × 10 mm × 55 mm, and tensile specimens were plate-shaped with a length of 130 mm.

Mechanical property tests primarily included impact toughness testing and tensile testing. For impact performance tests, specimens were prepared in accordance with GB/T 229-2020 [25] Metallic materials—Charpy pendulum impact test method. Impact tests were conducted using a PIT602H-4 pendulum impact testing machine (Wance, Shenzhen, China) at test temperatures of room temperature, −60 °C, −120 °C, and −196 °C. Three parallel specimens were tested under each condition, and the final results were taken as the average value.

For tensile tests, specimens were prepared according to GB/T 228.3-2019 [26] Metallic materials—Tensile testing—Part 3: Method of test at low temperature. Tensile tests were performed using a TSE504C universal testing machine (Wance, Shenzhen, China) equipped with a GWLS06 high/low-temperature tensile fixture, at room temperature and at −196 °C. The specimens were plate-type tensile samples with a gauge length of L_0_ = 25 mm, a width of 25 mm, and a thickness of 2 mm. A unified strain rate of 0.15 mm/s was applied. An axial extensometer with a 25 mm gauge length was employed to record the displacement of the gauge section in real time. For the −196 °C tests, the specimen fitted with the extensometer was fully immersed in liquid nitrogen and held for at least 15 min; it was then pulled to fracture immediately after a thermocouple attached to the specimen surface confirmed that thermal equilibrium had been reached. Throughout the test, the temperature was maintained constant at −196 °C in the liquid nitrogen bath. Parameters including elongation after fracture, ultimate tensile strength, and yield strength were measured, and the tensile fracture surfaces were also analysed by SEM. Three parallel specimens were tested under each condition to ensure the accuracy of the results.

Microstructural characterization was carried out via scanning electron microscopy (FE-SEM, AURIGA, iss, Oberkochen, Germany) and electron backscatter diffraction (EBSD, C-Nano+, Oxford Instruments, High Wycombe, UK), with energy-dispersive spectroscopy (EDS, Genesis 2000, EDAX, Mahwah, NJ, USA) employed for elemental analysis. The EBSD scan area was 267 μm × 183.6 μm. The statistical characterization of inclusions was conducted using a Thermo Scientific SEM (SEM/EDS, Explorer 4, Thermo Fisher Scientific (formerly FEI), Hillsboro, OR, USA)equipped with EDS and Perception automatic inclusion analysis software. Operating at 15 keV and 300× magnification, the system scanned a total area of 50 mm^2^ per specimen across 450 fields of view. Inclusions were identified from Backscattered Electron (BSE) images based on a minimum detection threshold of Equivalent Circular Diameter (ECD) ≥ 1 μm. The chemical composition of detected inclusions was subsequently determined via EDS for classification (e.g., Cr, MnS, and Ca-Si-Al types).

## 3. Results

### 3.1. Effect of Trace Nb on Tensile Properties

#### 3.1.1. Tensile Properties

**Figure 1:**
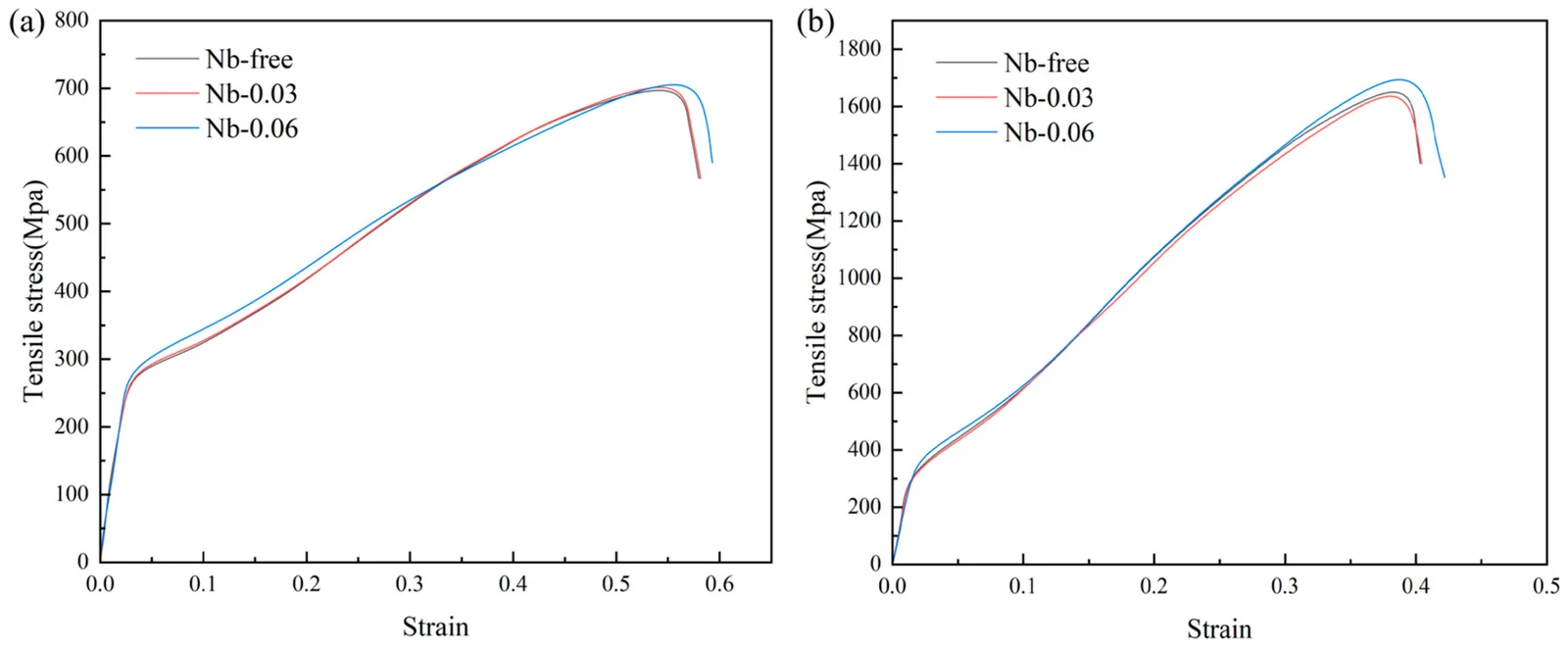
Tensile curves of 304 austenitic stainless steel with different Nb contents: (**a**) 25 °C; (**b**) −196 °C.

Figure 1 presents the engineering stress–strain curves of the experimental steels with different Nb contents at room temperature and −196 °C, and the corresponding tensile properties obtained after data processing are summarized in Table 2. As shown in Figure 1a, all three curves at room temperature display typical work-hardening features of austenitic stainless steel: after yielding, stress rises continuously with strain without a distinct yield plateau. The work-hardening regime extends over a large strain range, and necking initiates at a strain of approximately 0.55. Overall, the three curves closely overlap across most of the strain range, separating slightly only beyond a strain of ~0.50. Here, the 0.06 wt.% Nb steel shows marginally higher stress and enters necking first. This indicates that Nb addition has a limited effect on the slope of the work-hardening stage; its strengthening effect is mainly manifested as an increase in yield strength while retaining uniform plastic deformation capability. The 0.06 wt.% Nb steel achieves the highest tensile strength (704 MPa) and yield strength (274 MPa), which are 0.9% and 5.0% higher than those of the Nb-free steel (698 MPa, 261 MPa), respectively. Its elongation after fracture reaches 52.37%, comparable to or even slightly greater than that of the Nb-free steel. These results demonstrate that Nb microalloying can strengthen 304 stainless steel without sacrificing ductility, thereby mitigating the room-temperature strength–ductility trade-off to a certain extent.

As shown in Figure 1b and Table 2, the ultimate tensile strength at −196 °C exceeds twice that at room temperature, the work-hardening rate rises substantially, and necking occurs, followed by a rapid stress drop. In terms of curve morphology, the three curves show obvious fan-shaped divergence within the post-yield transition region (strain ≈ 0.05–0.10). Within this strain interval, the stress of the 0.06 wt.% Nb steel is higher than those of the Nb-free and 0.03 wt.% Nb steels, while the latter two nearly fully overlap. Upon entering the main work-hardening regime, the curves converge again. Near the peak stress, the 0.06 wt.% Nb steel once more exhibits a marginal lead, whereas the Nb-free and 0.03 wt.% Nb steels are barely distinguishable. This implies that the strengthening effect of Nb at low temperature concentrates primarily in the early deformation stage, and 0.03 wt.% Nb is insufficient to produce a measurable strengthening response. Quantitatively, the 0.06 wt.% Nb steel has a tensile strength of 1688 MPa and a yield strength of 350 MPa, representing respective increases of 1.1% and 11.1% relative to the Nb-free steel (1670 MPa, 315 MPa). Its elongation after fracture also remains the highest at 36.43%, compared with 34.99% for the Nb-free steel. Notably, the tensile strength of the 0.03 wt.% Nb steel (1666 MPa) is slightly lower than that of the Nb-free steel, indicating that low-temperature strength does not vary monotonically with Nb content. Even under cryogenic conditions, Nb enables strengthening with retained ductility. Within the Nb composition range investigated in this work, this beneficial effect is most pronounced in the 0.06 wt.% Nb steel.

Deformation-induced α′-martensite transformation may occur during deformation, and its contribution to work hardening and strength at −196 °C cannot be neglected. Because the present study did not quantitatively characterize its content, the portions of the aforementioned strength increase attributable to Nb microalloying and to deformation-induced martensite cannot be strictly separated; relevant quantitative conclusions await verification in subsequent work.

#### 3.1.2. Tensile Fracture Morphology

**Figure 2:**
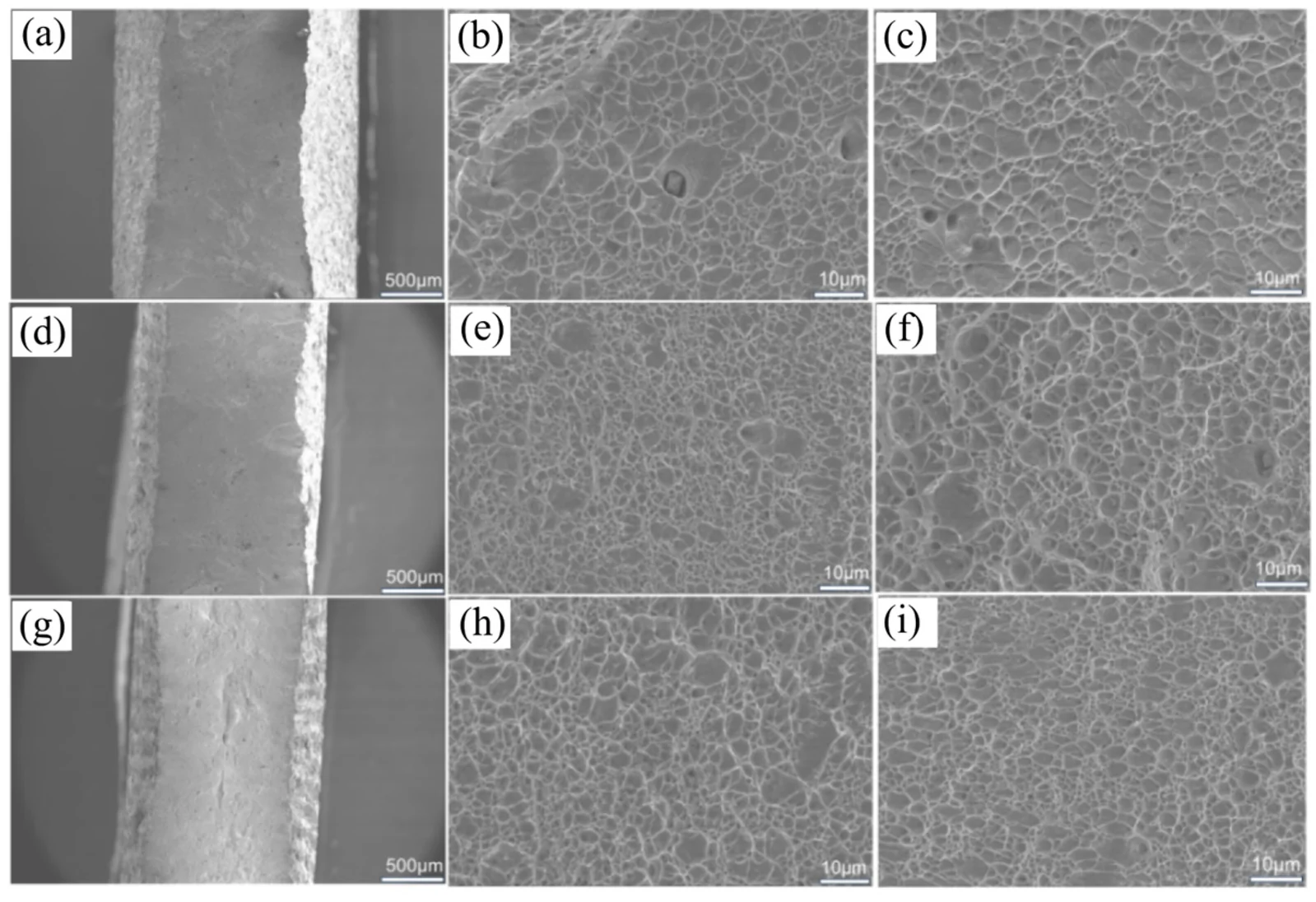
Room-temperature tensile fracture morphologies of 304 austenitic stainless steels with different Nb contents: (**a**–**c**) Nb-free; (**d**–**f**) Nb-0.03; (**g**–**i**) Nb-0.06.

Room-temperature tensile fracture morphologies (Figure 2) revealed that all specimens exhibited typical ductile fracture characteristics on the macroscopic scale, comprising a fibrous zone, a radial zone, and shear lips. However, the Nb content exerted a pronounced regulatory effect on the morphology of each region. The Nb-free specimen (Figure 2a) showed irregular edges of the fibrous zone, disordered radial zone textures, and uneven shear lip widths, indicating insufficient plastic deformation coordination. In contrast, the Nb-0.06 specimen (Figure 2g) exhibited the best overall fracture flatness, with uniformly distributed fibrous zones and well-defined shear lips at 45° to the tensile direction, suggesting a more stable plane-stress shear-type fracture process. With increasing Nb content, the macroscopic fracture morphology transitioned from rough and non-uniform to flat and uniform. The Nb-0.03 specimen (Figure 2d) served as an intermediate state, with improved fibrous zone roughness and enhanced shear lip continuity, confirming the gradient optimization effect of Nb on stress distribution during fracture.

**Figure 3:**
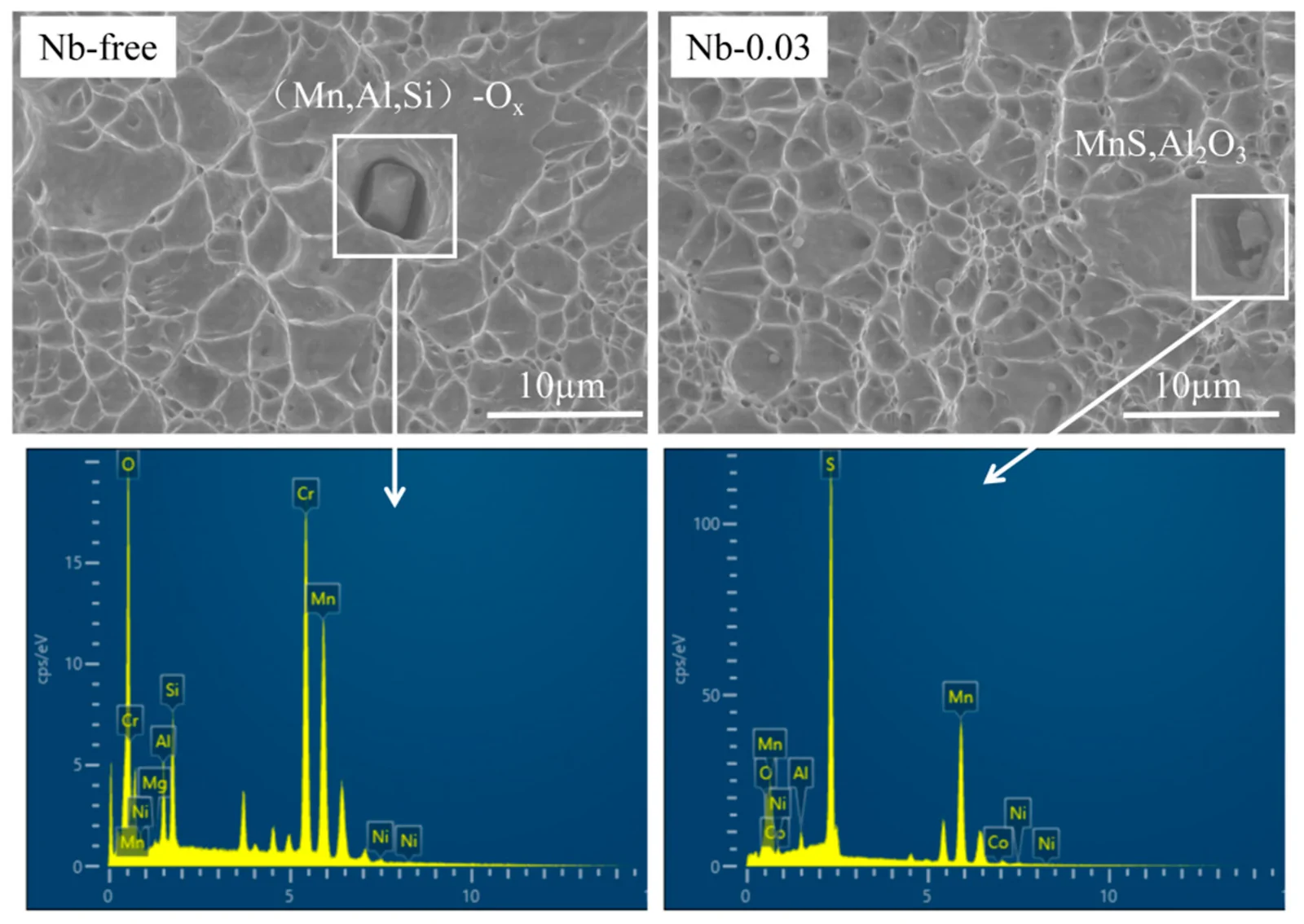
EDS maps of room-temperature tensile fracture surfaces of 304 austenitic stainless steels with different Nb contents.

At the microscale, the Nb-free steel fracture surface (Figure 2b,c) contained small, unevenly distributed dimples, and coarse (Al, Mn, Si)–O_x_ inclusions (Figure 3) were visible within the dimples. These inclusions act as preferential sites for microvoid nucleation, readily inducing local stress concentration. After Nb addition, finer and more regular tear ridges appeared (Figure 2e,f,h,i).

**Figure 4:**
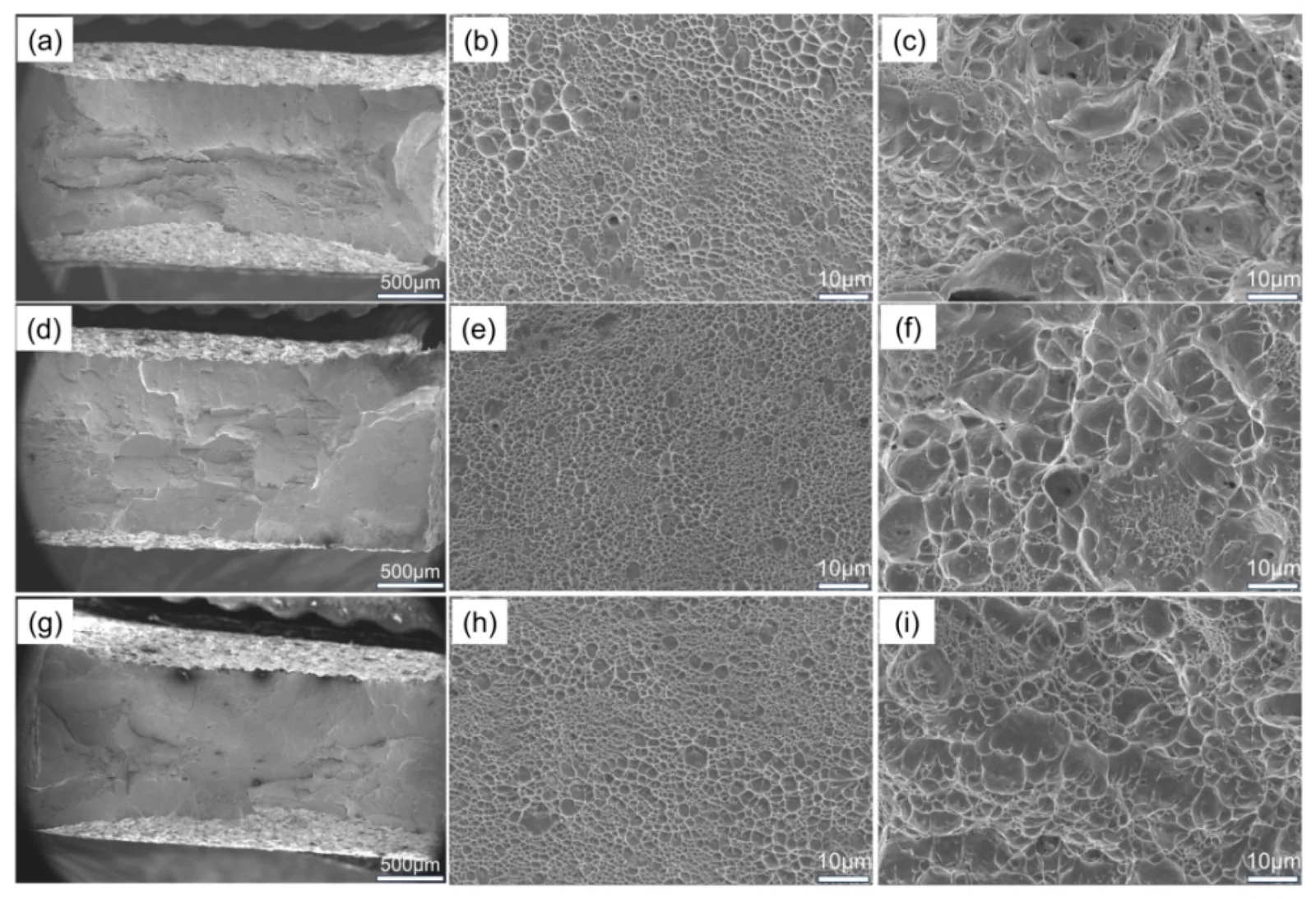
Tensile fracture morphologies at −196 °C of 304 austenitic stainless steels with different Nb contents: (**a**–**c**) Nb-free; (**d**–**f**) Nb-0.03; (**g**–**i**) Nb-0.06.

At the cryogenic temperature of −196 °C (Figure 4), the macroscopic plastic deformation zones—namely, the fibrous zone and the shear lip—were markedly reduced in all tested steels, indicating a pronounced embrittlement tendency. Microscopically, a mixed fracture mode of dimples coexisting with cleavage facets was observed, suggesting that the low temperature suppressed dislocation slip and thereby reduced the resistance to crack propagation. Notably, the proportion of cleavage regions in the Nb-bearing steels was significantly lower than that in the Nb-free steel, with extensive dimple-dominated zones remaining predominant. This composition-dependent fracture morphology is consistent with the retardation of the low-temperature ductile-to-brittle transition by Nb addition. It should be noted that the presence of cleavage facets is compatible with the effects of strain-induced α′-martensitic transformation; however, fracture morphology alone cannot confirm the presence or quantify the content of α′-martensite. Owing to experimental limitations, the present study did not characterize it quantitatively, and therefore the specific contribution of α′-martensite to the increased yield strength of the Nb-0.06 steel cannot be assessed on this basis.

### 3.2. Effect of Trace Nb on Impact Properties

#### 3.2.1. Impact Properties

**Figure 5:**
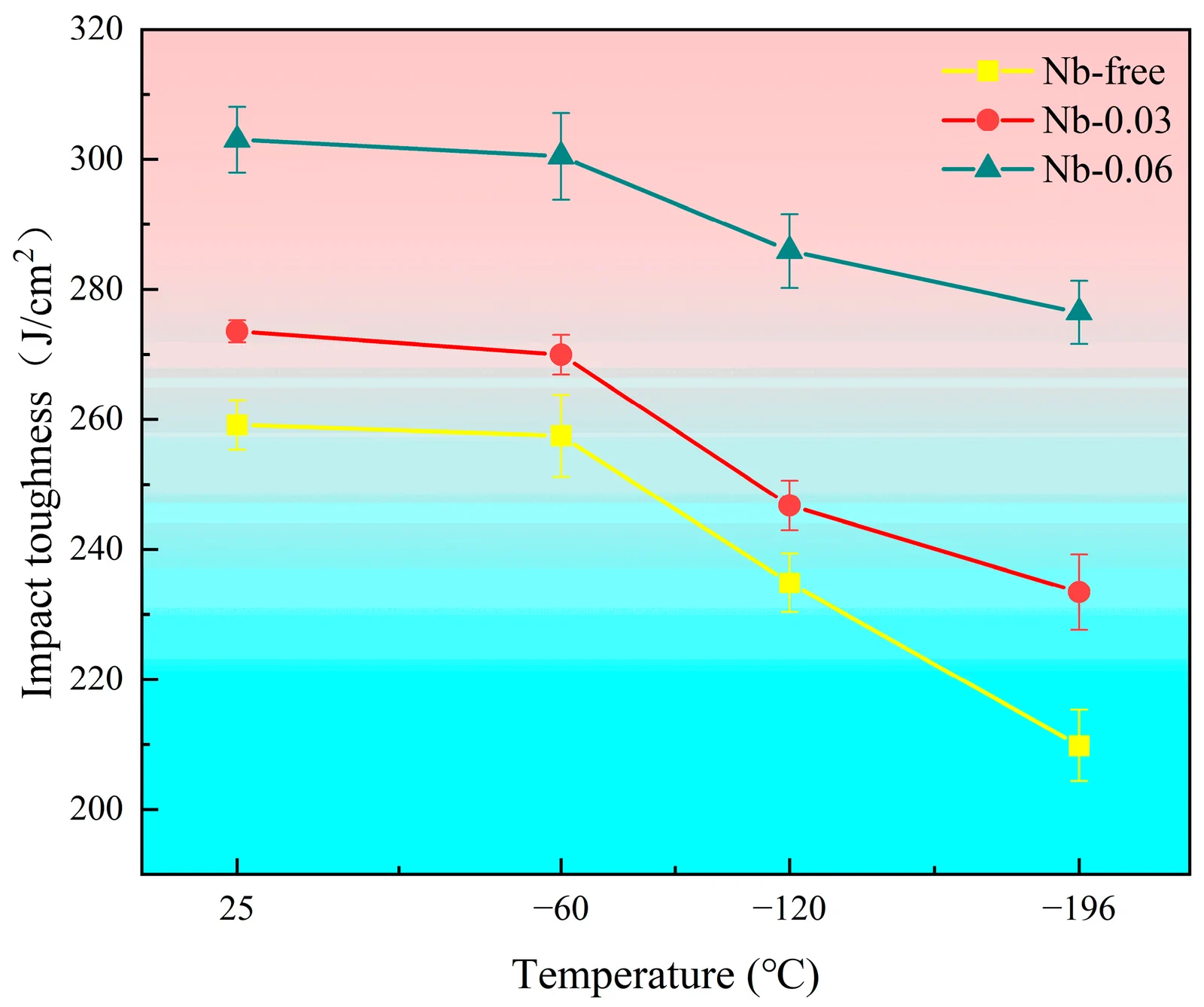
Impact test results of 304 austenitic stainless steels with different Nb contents.

Figure 5 and Table 3 show the impact toughness values of the experimental steels at various temperatures. The impact toughness of all specimens decreased with decreasing test temperature, which is an inevitable consequence of reduced atomic thermal activation energy and increased resistance to dislocation slip at low temperatures, leading to diminished plastic deformation capacity and reduced energy absorption for crack propagation, ultimately resulting in lower impact toughness. Nevertheless, at the same temperature, impact toughness increased notably with increasing Nb content. Especially at −196 °C, the impact toughness of Nb-0.06 steel (276.48 J/cm^2^) was 31.8% higher than that of Nb-free steel (209.84 J/cm^2^) and 18.4% higher than that of Nb-0.03 steel (233.48 J/cm^2^). This indicates that Nb microalloying effectively suppresses low-temperature embrittlement, and its beneficial effect becomes more pronounced as temperature decreases [27].

#### 3.2.2. Impact Fracture Morphology

**Figure 6:**
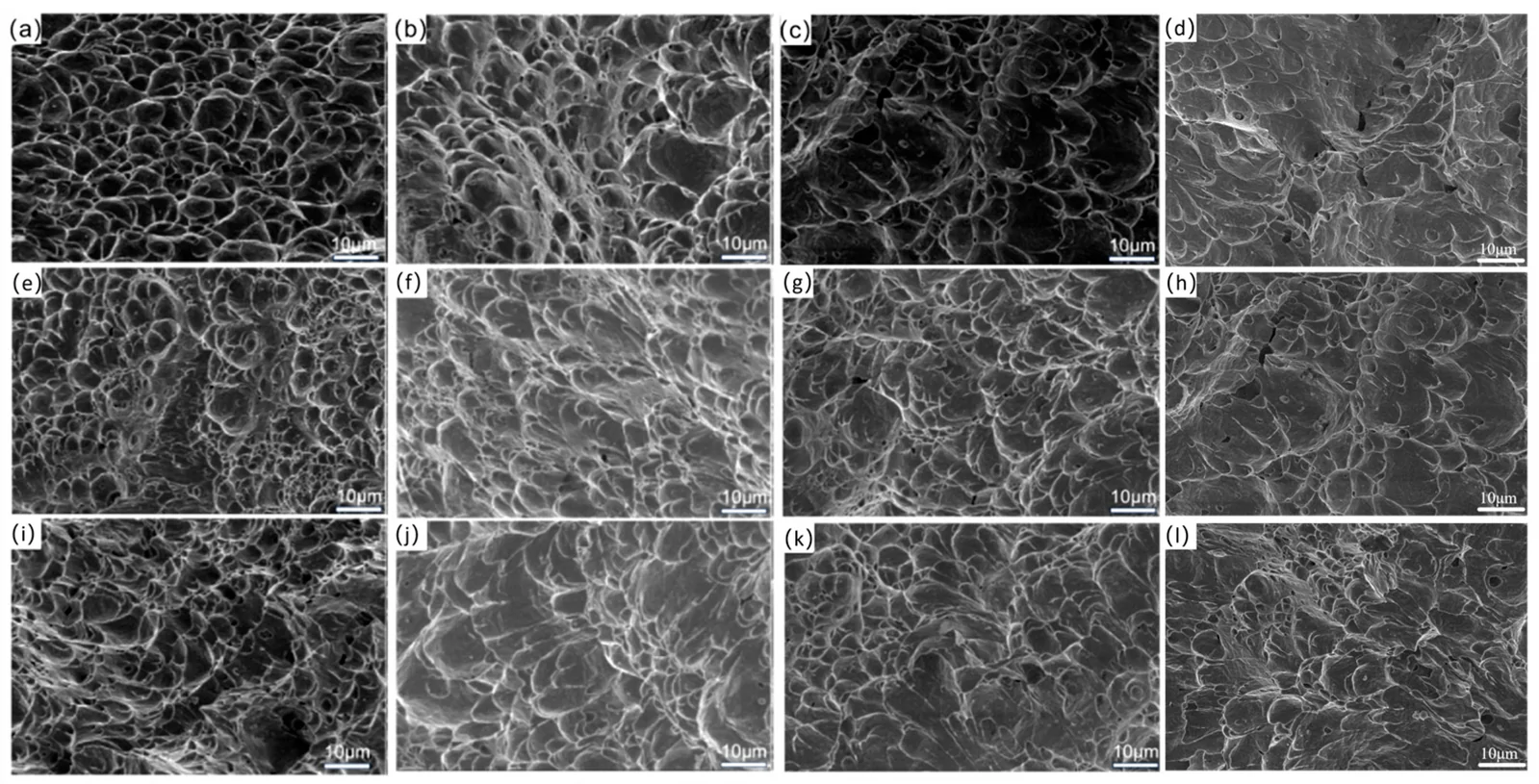
SEM morphologies of impact fracture surfaces of experimental steels at different temperatures: (**a**–**d**) Nb-free; (**e**–**h**) 0.03 wt.% Nb; (**i**–**l**) 0.06 wt.% Nb; (**a**,**e**,**i**) RT; (**b**,**f**,**j**) −60 °C; (**c**,**g**,**k**) −120 °C; (**d**,**h**,**l**) −196 °C.

As the test temperature decreased from room temperature to −196 °C, the impact fracture morphologies of all three steel groups exhibited a transition from ductile to brittle fracture. At room temperature and −60 °C (Figure 6), all the impact fracture surfaces were predominantly ductile, displaying a typical dimpled morphology. The impact dimples at room temperature were mostly circular or elliptical pits, with more pronounced traces of plastic deformation and a more complete microvoid coalescence process. At −60 °C, the reduced thermal activation energy of the atoms and the increased resistance to dislocation slip weakened the plastic deformation capacity; consequently, the dimples were generally smaller and shallower, and some regions became flatter.

When the temperature dropped to −120 °C, macrocracks and a cleavage tendency appeared on the fracture surface of the Nb-free steel, indicating that it had entered the ductile-to-brittle transition region. The Nb-0.03 steel showed fine notches and locally flattened zones, whereas the Nb-0.06 steel still exhibited a predominantly dimpled morphology, with flat regions occurring only locally. At −196 °C, the Nb-free steel displayed a mixed dimple–cleavage morphology, the Nb-0.03 steel was dominated by fine and dense dimples, and the Nb-0.06 steel retained a relatively uniform dimpled morphology. These observations indicate that Nb microalloying suppresses low-temperature embrittlement, shifting the ductile-to-brittle transition temperature of the Nb-0.06 steel below the temperature range examined in this study.

### 3.3. Effect of Nb Microalloying on Microstructural Refinement and Mechanical Properties

The mechanical-property and fracture-surface analyses presented above indicate that Nb microalloying improves the overall mechanical performance of 304 austenitic stainless steel over a wide temperature range, and that this improvement shows a clear correlation with the evolution of both the grain size and the inclusion population. It should be noted that the existing data are insufficient to attribute the performance enhancement predominantly to any single mechanism; the following discussion therefore addresses grain refinement and inclusion modification separately.

#### 3.3.1. Properties Grain Refinement

**Figure 7:**
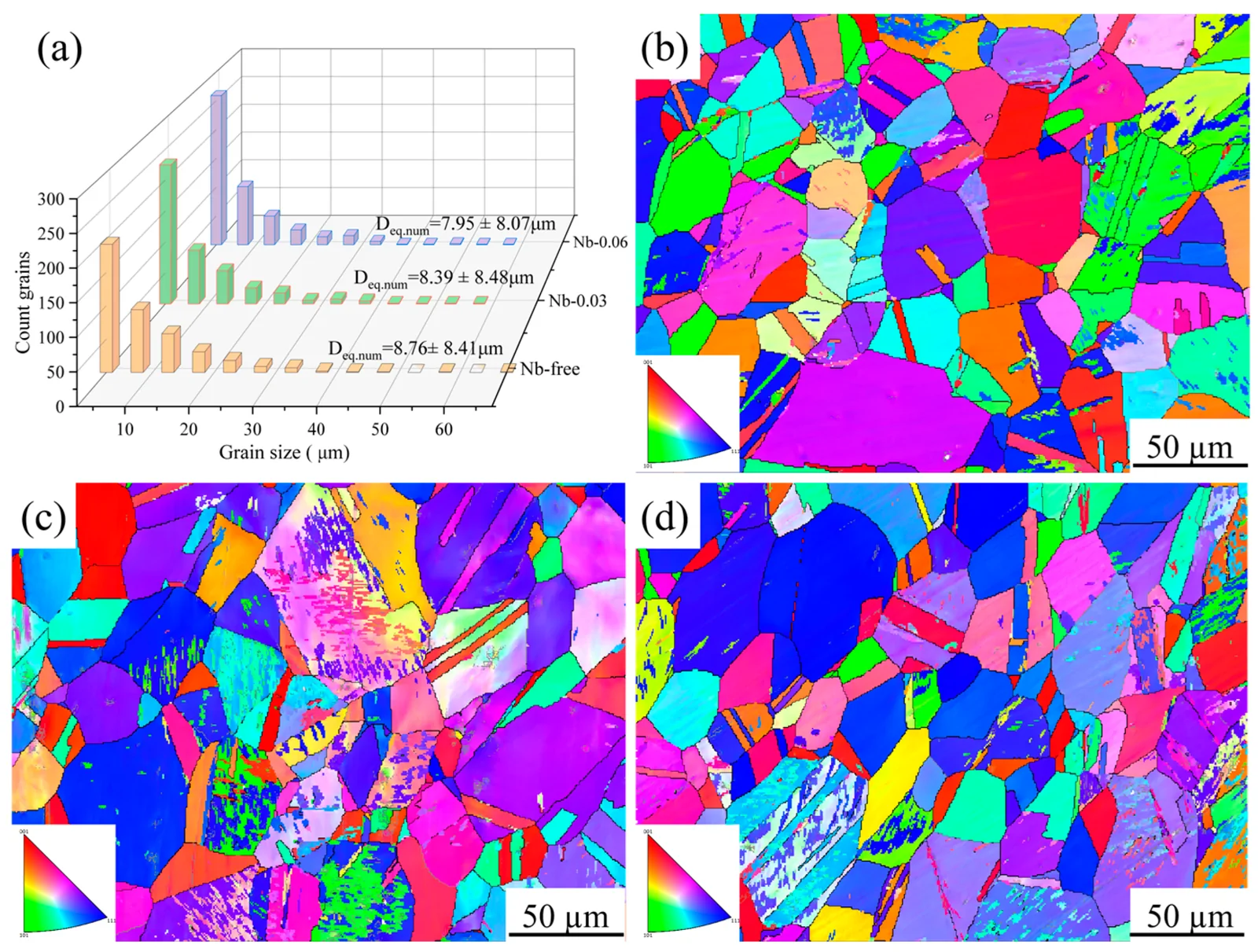
EBSD-IPF maps of 304 austenitic stainless steel samples with different Nb contents: (**a**) grain size distributions of the Nb-free, Nb-0.03, and Nb-0.06 steels. The values are presented as mean ± standard deviation. (**b**) Nb-free. (**c**) Nb-0.03. (**d**) Nb-0.06.

The EBSD-IPF maps (Figure 7) reveal that, as the Nb content increases from 0 to 0.06 wt.%, the numbers of statistically valid grains collected from the three specimens are 405, 421, and 442, respectively. As shown in Figure 7a, the grain-size distributions of all three specimens are markedly right-skewed (positively skewed), with their modes located in the finest size bin. With increasing Nb content, the population of ultrafine grains increases while the fraction of coarse grains declines, and the entire distribution shifts progressively toward smaller sizes, indicating a gradual enhancement of grain refinement. At a Nb addition of 0.06 wt.%, the number-averaged grain size decreases from 8.76 ± 8.41 μm (Nb-free) to 7.95 ± 8.07 μm, corresponding to a relative reduction of 9.25%; the Nb-0.03 specimen exhibits an intermediate value of 8.39 ± 8.48 μm. According to the Hall–Petch relationship, grain refinement can simultaneously enhance strength and toughness.

*σ_y_* = *σ*_0_ + *k_H_d*^−1/2^ (1)

The terms in Equation (1) are defined as follows: *σ_y_*—yield strength (MPa); *σ*_0_—lattice friction stress; *k_H_*—Hall–Petch slope (MPa·μm^−1/2^); and *d*—average grain size (μm).

Using kH = 632.6 MPa·μm^−1/2^ [28], applicable to 304L austenitic stainless steel over a broad grain-size range, the grain-refinement contribution associated with a grain size refined from 8.76 μm to 7.95 μm is calculated to be about 10.6 MPa. Given that the Hall–Petch coefficient is only weakly temperature-dependent, this term is essentially constant from room temperature to cryogenic temperatures, consistent with the ~13 MPa room-temperature increment being largely governed by grain refinement. The remaining ~24.4 MPa increment at −196 °C, however, exceeds what can be explained by grain refinement alone. Nb solute–dislocation interactions offer a likely explanation: at cryogenic temperatures, suppressed thermal activation prevents dislocation motion from overcoming obstacles with the aid of thermal fluctuations, so that the drag resistance exerted by dissolved Nb atoms—and potentially by Nb–interstitial complexes—is markedly amplified [29]. Strain-induced α′-martensitic transformation (TRIP) may also contribute, but in the absence of quantitative characterization of the martensite content before and after deformation, the contributions of solute drag and TRIP cannot be decoupled. Thus, grain refinement and solute–dislocation interactions are the leading candidates for the cryogenic yield-strength enhancement, although their precise partitioning—and that of the transformation contribution—awaits future investigation.

#### 3.3.2. Inclusion Evolution as an Accompanying Phenomenon

**Figure 8:**
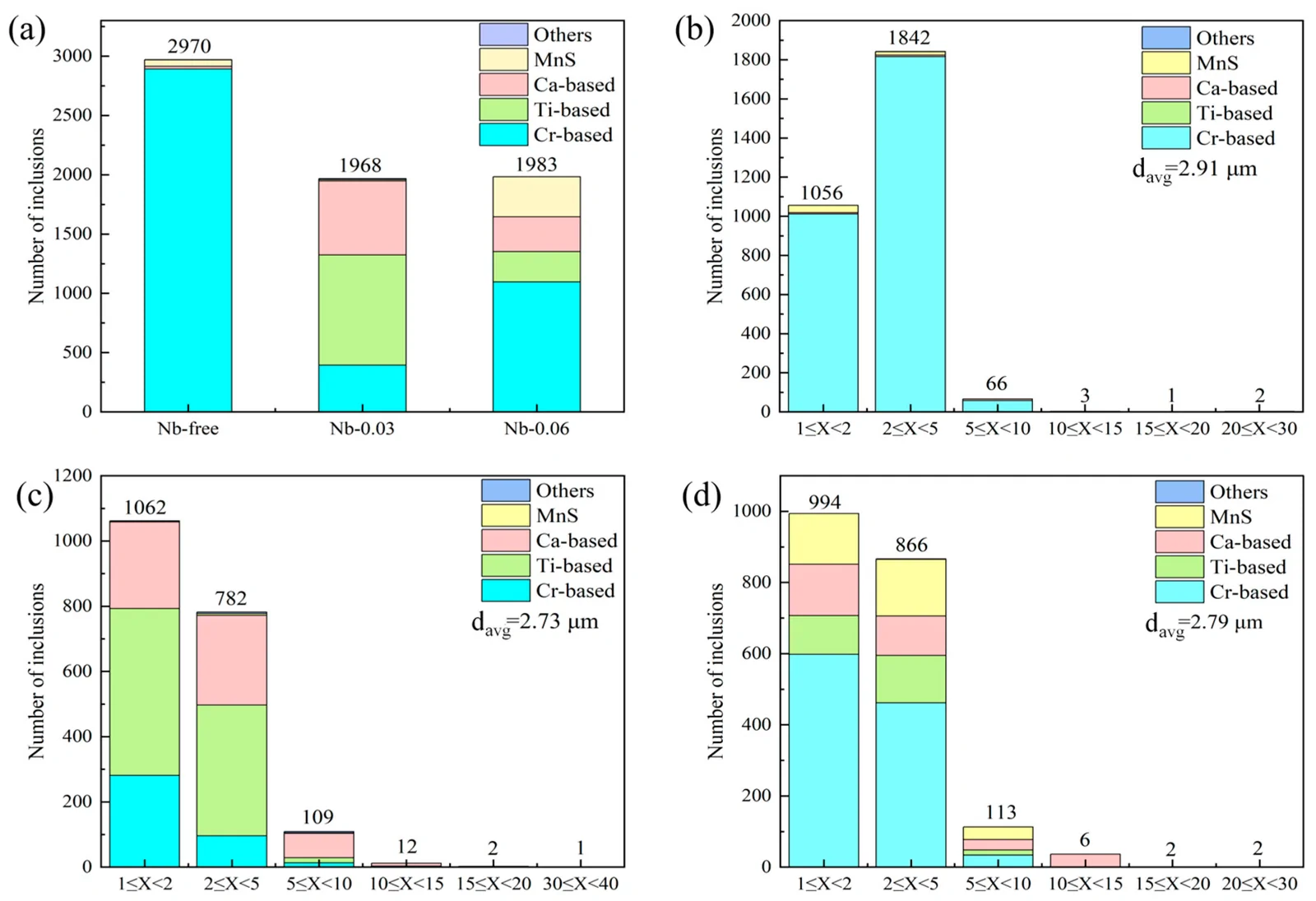
Types, number, and size distribution of inclusions in 304 austenitic stainless steels with different Nb contents: (**a**) types and total number of inclusions; (**b**) Nb-free steel; (**c**) Nb-0.03 steel; (**d**) Nb-0.06 steel.

**Figure 9:**
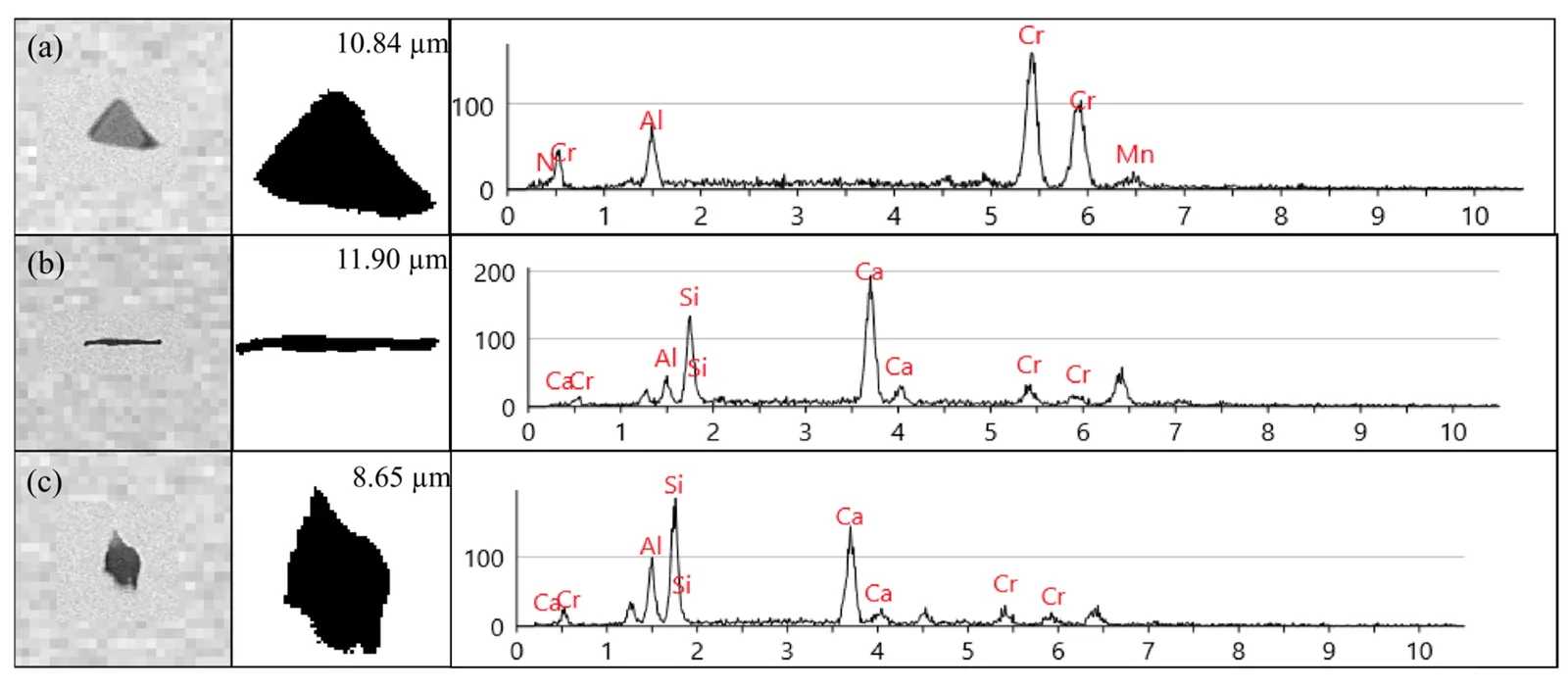
SEM morphologies, shape outlines, and EDS spectra of typical inclusions in 304 austenitic stainless steels with different Nb contents: (**a**) Nb-free steel; (**b**) Nb-0.03 steel; (**c**) Nb-0.06 steel.

Upon Nb addition, the type, size, and morphology of the inclusion population changed markedly (Figure 8 and Figure 9), and the evolution characteristics were closely correlated with the Nb content, exhibiting a distinct stage-like dependence.

In the Nb-0.03 specimen, the total inclusion number density decreased from 59.4 particles/mm^2^ in the Nb-free steel to 39.36 particles/mm^2^, while the weight-averaged size decreased from 2.91 μm to 2.73 μm; correspondingly, the inclusion population shifted from being predominantly Cr–Mn-based inclusions to predominantly complex Ti–Ca–Al–Si deoxidation products [30]. This indicates that Nb addition is closely associated with the evolution of the inclusion population and may have influenced the conditions governing inclusion formation and evolution. However, at this low Nb level the modification effect remained incomplete: in addition to numerous complex inclusions, one calcium aluminate particle of approximately 31.27 μm and many high-aspect-ratio elongated Ca–Si–Al complex inclusions remained (Figure 9b). The persistence of these coarse, hard, brittle, and elongated high-risk inclusions within the steel matrix is most likely attributable to their incomplete removal during collision, agglomeration, and floatation, and they may still serve as preferential sites for crack initiation, limiting any further improvement in low-temperature impact toughness.

When the Nb content was increased to 0.06 wt.%, the inclusion modification exhibited different characteristics. The total inclusion number density was 39.66 particles/mm^2^ and the weight-averaged size was 2.79 μm, both of which were close to those of the Nb-0.03 specimen. In terms of inclusion composition, however, the proportion of Cr-based inclusions increased somewhat, whereas that of Ti–Ca–Al-based deoxidation products decreased markedly. Concurrently, the number of harmful inclusions larger than 10 μm decreased from 15 to 10, no inclusion larger than 30 μm was detected, highly elongated inclusions of high aspect ratio almost completely disappeared, and the inclusions became finer, more globular, and more uniformly distributed overall. These changes indicate that, at the higher Nb level, inclusion modification manifested primarily in the elimination of large, high-risk inclusions, globularization of the morphology, and homogenization of the distribution [31], rather than in any significant change in the total inclusion count or the average inclusion size.

It should be noted that although the evolution of inclusion characteristics with Nb content coincides with the variation trend of low-temperature impact toughness, the existing data are insufficient to attribute the toughness improvement predominantly to inclusion modification. On the one hand, the Nb-free specimen, which had the lowest numbers of inclusions larger than 5 μm and 10 μm, nevertheless exhibited the poorest Charpy impact energy at −196 °C. On the other hand, the Nb-0.03 and Nb-0.06 specimens were nearly identical in inclusion number density and average inclusion size, yet differed by approximately 18% in impact energy, indicating that minor differences in inclusion number and average size alone can hardly account for the significant change in toughness. In addition, the present EDS results detected no Nb-bearing inclusions, demonstrating that under the present experimental conditions Nb did not directly participate in the precipitation process of inclusions by forming either Nb-containing inclusions or Nb–Ti complex inclusions. Taken together, the evolution of inclusions toward a finer, more globular, and more dispersed distribution is an accompanying phenomenon following Nb addition, and may to some extent have reduced the stress concentration and the tendency for microcrack initiation associated with coarse, hard, brittle inclusions. The marked improvement in low-temperature impact toughness, however, is more plausibly attributed to the strengthening effect of solute Nb on the austenite matrix—namely, grain refinement, solid-solution strengthening, and grain-boundary segregation. This interpretation is more consistent with the available experimental evidence, although a quantitative correspondence between inclusion characteristics and toughness still requires further investigation.

## 4. Conclusions

Within the investigated Nb content range (0, 0.03, and 0.06 wt.%), the addition of 0.06 wt.% Nb produced the largest measured response and improved the strength, ductility, and impact toughness of 304 austenitic stainless steel from room temperature to −196 °C. Compared with the Nb-free steel, the room-temperature yield strength increased by approximately 5% and the elongation after fracture by approximately 2.4%; at −196 °C, the yield strength increased by 11.1%, the elongation after fracture by 4.1%, and the impact toughness by 31.8%. These results indicate that Nb microalloying can mitigate the strength–ductility trade-off at cryogenic temperatures to a certain extent.

Following Nb addition, both the grain size and the inclusion population underwent regular evolution. As the Nb content increased from 0 to 0.06 wt.%, the statistically determined average grain size decreased from 8.76 μm to 7.95 μm, a reduction of approximately 9.25%. Concurrently, the inclusion population shifted from being predominantly Cr–Mn-based inclusions to predominantly complex Ti–Ca–Al–Si deoxidation products, while the number of large and elongated harmful inclusions decreased and their distribution became more dispersed. The strength increment of approximately 13 MPa at room temperature is comparable to the grain-refinement contribution of approximately 10.6 MPa. Of the approximately 35 MPa increase in yield strength at −196 °C, however, grain refinement accounts for only about 10.6 MPa, and the remaining portion cannot be explained by grain refinement alone. Moreover, the Nb-0.03 and Nb-0.06 specimens are fairly close in grain size, inclusion number density, and weight-averaged inclusion size, yet their yield strengths at −196 °C differ by approximately 18% (316 MPa versus 350 MPa). This indicates that differences in grain refinement and inclusion distribution characteristics alone are insufficient to account for the pronounced cryogenic strengthening response.

A limitation of this study is the lack of quantitative characterization of α′-martensite content during deformation. Consequently, the individual relative contributions of Nb-induced grain refinement, solid-solution strengthening, and deformation-induced α′-martensite transformation to the cryogenic mechanical response could not be fully differentiated. Future work will quantitatively investigate the functional relationship between α′-martensite evolution and Nb content, temperature, and strain.

This study confirms that Nb microalloying (0.06 wt.%) can enhance the toughness reserve and fracture resistance of stainless steel at extreme cryogenic temperatures without substantial cost increase, providing experimental evidence and alloy design references for its potential application in extreme cryogenic engineering structures such as LNG storage and transportation, liquid hydrogen tanks, and superconducting magnet supports. However, it should be noted that the tests were performed at −196 °C, which does not fully represent liquid hydrogen storage conditions; therefore, the results of this study provide a basis for further research rather than direct confirmation of applicability.

## Figures and Tables

**Table 1:** Chemical compositions of 304 austenitic stainless steels with different Nb contents (wt.%).

Steel	C	Cr	Mn	Si	Ni	S	N	Al	P	Cu	Mo	Nb	Fe
Nb-free	0.027	18.064	0.837	0.345	7.816	0.002	0.032	0.004	0.024	0.024	0.006	-	Bal.
Nb-0.03	0.022	18.129	0.841	0.321	7.843	0.002	0.031	0.005	0.023	0.050	0.008	0.03	Bal.
Nb-0.06	0.026	18.291	0.811	0.345	7.841	0.003	0.036	0.005	0.021	0.034	0.005	0.06	Bal.

**Table 2:** Tensile test results of 304 austenitic stainless steels with different Nb contents.

Temperature (°C)	Steel	Yield Strength (MPa)	Ultimate Tensile Strength (MPa)	Elongation After Fracture (%)
25	Nb-free	261.20 ± 1.52 (a)	698.11 ± 2.36 (a)	52.37 ± 0.52 (a)
	Nb-0.03	265.01 ± 1.73 (ab)	699.32 ± 1.89 (a)	52.74 ± 0.67 (a)
	Nb-0.06	274.00 ± 2.02 (b)	703.89 ± 3.21 (a)	53.65 ± 0.82 (a)
−196	Nb-free	315.35 ± 0.88 (a)	1670.00 ± 2.00 (a)	34.99 ± 0.61 (a)
	Nb-0.03	316.00 ± 2.65 (a)	1665.98 ± 3.33 (a)	35.15 ± 0.77 (a)
	Nb-0.06	350.34 ± 1.66 (b)	1688.88 ± 4.24 (b)	36.43 ± 0.48 (a)

Note: Tensile data are presented as mean ± standard deviation (*n* = 3). Within each column, values sharing different letters indicate statistically significant differences (*p* < 0.05; one-way ANOVA followed by Tukey’s HSD test), whereas values with the same letter indicate no significant difference. The Nb-0.03 group and the Nb-free group showed no significant difference for any measured parameter, whereas the Nb-0.06 group differed significantly from the Nb-free group in the yield strength at 25 °C and in the yield strength and ultimate tensile strength at −196 °C. Full tensile test results for the 304 austenitic stainless steels with different Nb contents are provided in Supplementary Table S1, and the corresponding one-way ANOVA and Tukey’s HSD post-hoc test results are provided in Supplementary Table S2.

**Table 3:** Impact toughness of 304 austenitic stainless steels with different Nb contents at various temperatures.

Steel	Temperature (°C)	Impact Toughness (J/cm^2^)			Average (J/cm^2^)
Nb-free	RT	254.90	262.23	260.33	259.15 ± 3.80
	−60	261.23	250.20	260.98	257.46 ± 6.30
	−120	232.80	231.78	240.03	234.86 ± 4.50
	−196	212.48	213.53	203.53	209.84 ± 5.50
Nb-0.03	RT	271.65	274.03	274.93	273.54 ± 1.69
	−60	273.43	268.53	267.88	269.94 ± 3.03
	−120	244.78	251.20	244.38	246.79 ± 3.83
	−196	238.85	234.18	227.33	233.48 ± 5.79
Nb-0.06	RT	297.60	303.83	307.60	303.01 ± 5.05
	−60	298.95	294.68	307.73	300.45 ± 6.65
	−120	287.58	290.58	279.58	285.91 ± 5.69
	−196	281.20	271.50	276.73	276.48 ± 4.85

## Data Availability

The original contributions presented in this study are included in the article/Supplementary Material. Further inquiries can be directed to the corresponding authors.

## Supplementary Materials

The following supporting information can be downloaded at https://www.mdpi.com/article/10.3390/ma19214719/s1. Table S1: Tensile test results of 304 austenitic stainless steels with different Nb contents; Table S2: Results of one-way ANOVA and Tukey’s HSD post-hoc test.
