# Supplementary material for: Mechanistic Study on Nb Microalloying Synergistically Enhancing the Cryogenic Strength–Toughness of Austenitic Stainless Steel
Source: Materials (Basel). 2026 Nov 6;19(21):4719. doi: 10.3390/ma19214719 (PMC13765036; doi:10.3390/ma19214719)
Supplement: Supplementary file 1 [file materials-19-04719-s001.zip › materials-4548785-supplementary.pdf]

Supplementary Materials

Mechanistic Study on Nb Microalloying Synergistically Enhancing the Cryogenic Strength–Toughness of Austenitic Stainless Steels

Lei Huang[1,2], Riqing Huang[1,2], Qing Li[2,*],Xiangzhu Wu[2], Tingping Hou[2], Tianliang Zhao[2], Sundas Arshad[2], Marina Afzal[2], Faiqa Barkat[2], Kaiming Wu[2,*]

Table S1. Tensile test results of 304 austenitic stainless steels with different Nb contents.

Temperature (℃)	Steel	Yield strength(MPa)				Ultimate tensile strength (MPa)				Elongation after fracture (%)			
25℃	Nb-free	260.6	262.93	260.07	261.20±1.52	698.58	700.19	695.55	698.11±2.36	52.37	52.89	51.86	52.37±0.52
	Nb-0.03	265.39	266.51	263.12	265.01±1.73	699.59	701.06	697.31	699.32±1.89	52.98	53.27	51.98	52.74±0.67
	Nb-0.06	274.7	275.58	271.72	274.00±2.02	703.89	707.1	700.68	703.89±3.21	53.78	54.4	52.77	53.65±0.82
-196℃	Nb-free	315.75	315.96	314.34	315.35±0.88	1669.56	1672.18	1668.26	1670±2.00	34.69	35.70	34.59	34.99±0.61
	Nb-0.03	317.53	317.53	312.94	316.00±2.65	1665.98	1669.31	1662.65	1665.98±3.33	35.08	35.95	34.44	35.15±0.77
	Nb-0.06	351.24	351.36	348.42	350.34±1.66	1685.80	1692.76	1685.08	1687.88±4.24	36.43	36.91	35.96	36.43±0.48

Table S2 Results of one-way ANOVA and Tukey's HSD post-hoc test

Temperature (℃)	Property	F(2,6)	ANOVA p	η^2	Significant Tukey HSD pairs (adjusted p)
25℃	**Yield strength(MPa)**	41.41	0.0003	0.932	Nb-free vs Nb-0.06: 0.0009; Nb-0.03 vs Nb-0.06: 0.0054
25℃	**Ultimate tensile strength (MPa)**	4.31	0.069	0.590	-
25℃	**Elongation after fracture (%)**	2.77	0.140	0.480	-
-196℃	**Yield strength(MPa)**	341.24	＜0.0001	0.991	Nb-free vs Nb-0.06: <0.0001; Nb-0.03 vs Nb-0.06: <0.0001
-196℃	**Ultimate tensile strength (MPa)**	36.99	0.0004	0.925	Nb-free vs Nb-0.06: 0.0041; Nb-0.03 vs Nb-0.06: 0.0014
-196℃	**Elongation after fracture (%)**	4.75	0.058	0.613	-

Table note: One-way ANOVA was used to compare the tensile properties of specimens with three Nb contents(Nb-free, Nb-0.03, Nb-0.06) at 25℃ and -196℃, with rz = 3 independent replicates for each group. Levene's test confirmed homogeneity of variances (all p > 0.05). Post-hoc multiplee comparisons were performed using Tukey's HSD correction at significance level a = 0.05; reported p-values are adjussted. n2 denotes the effect size."--" indicates no statistically significant pairwise difference.
